# KIR in Allogeneic Hematopoietic Stem Cell Transplantation: Need for a Unified Paradigm for Donor Selection

**DOI:** 10.3389/fimmu.2022.821533

**Published:** 2022-02-15

**Authors:** Adèle Dhuyser, Alice Aarnink, Michaël Pérès, Jyothi Jayaraman, Neda Nemat-Gorgani, Marie Thérèse Rubio, John Trowsdale, James Traherne

**Affiliations:** ^1^ Histocompatibility Laboratory, CHRU de Nancy, Vandoeuvre-les-Nancy, France; ^2^ IMoPA6, UMR7365 CNRS, Université de Lorraine, Vandoeuvre-les-Nancy, France; ^3^ Department of Pathology, University of Cambridge, Cambridge, United Kingdom; ^4^ Department of Hematology, CHRU de Nancy, Vandoeuvre-les-Nancy, France

**Keywords:** killer immunoglobulin-like receptors (KIR), allogeneic hematopoietic stem cell transplantation (aHSCT), Donor selection, alloreactivity potential, predictive model

## Abstract

Allogeneic hematopoietic stem cell transplantation (aHSCT) is a lifesaving therapy for hematological malignancies. For years, a fully matched HLA donor was a requisite for the procedure. However, new immunosuppressive strategies have enabled the recruitment of viable alternative donors, particularly haploidentical donors. Over 95% of patients have at least two potential haploidentical donors available to them. To identify the best haploidentical donor, the assessment of new immunogenetic criteria could help. To this end, the clinical benefit of KIR genotyping in aHSCT has been widely studied but remains contentious. This review aims to evaluate the importance of KIR-driven NK cell alloreactivity in the context of aHSCT and explain potential reasons for the discrepancies in the literature. Here, through a non-systematic review, we highlight how the studies in this field and their respective predictive models or scoring strategies could be conceptually opposed, explaining why the role of NK cells remains unclear in aHCST outcomes. We evaluate the limitations of each published prediction model and describe how every scoring strategy to date only partly delivers the requirements for optimally effective NK cells in aHSCT. Finally, we propose approaches toward finding the optimal use of KIR genotyping in aHSCT for a unified criterion for donor selection.

## 1 Introduction

Allogeneic hematopoietic stem cell transplantation (aHSCT) is a lifesaving therapy for hematological as well as non-hematological diseases (1, 2). Technological advances notably in the field of molecular biology and drug development have enabled personalized approaches, improving the management of negative outcomes such as graft versus host disease (GVHD) or graft failure. A major step forward in aHSCT has been the possibility of using alternative donors, a feature that has increased markedly this past decade (3).

For years, it was thought that a fully matched HLA donor, i.e., a donor with 10/10 allele compatibility for the HLA-A, HLA-B, HLA-C, HLA-DRB1, and HLA-DQB1, loci was required for aHSCT. Considering the average number of siblings in Caucasian populations, the likelihood of finding a matched sibling donor (MSD) is around 30% (4), but this probability increases in populations with higher natality rates. The likelihood of finding a matched unrelated donor (MUD) depends on the ethnicity and is much higher for whites of European descent than minorities such as blacks of South or Central American descent, 75% vs. 16% respectively, according to US registries (5, 6). Moreover, MUDs are only available within a median time of 2–3 months from the start of the donor search, a delay that might be too long for patients requiring aHSCT for high-risk hematological malignancies (7).

## 2 Paradigm Shift in Donor Availability and Donor Selection

### 2.1 Alternative Donors for aHSCT

When no fully matched HLA donor is available or can be recruited within an acceptable timescale, aHSCT can therefore be performed with mismatched unrelated donors (MMUD), umbilical blood cord (UBC) stem cell products, or haploidentical (haplo-) related donors. Each of these graft sources tend to lead to particular clinical outcomes and have specific pros and cons arising from transplantation logistics (8–10) (**Supplementary Table 1**).

Concerning haplo-HSCT, the limiting factor of success in the past was the T-cell response of the donor to allogeneic HLA molecules resulting in high incidences of GVHD and unacceptable treatment-related mortality (TRM) (11–13). T-cell depletion (TCD) has, therefore, revolutionized haplo-HSCT clinical outcomes. Currently, there are several different strategies to obtain TCD (**Table 1**). A large retrospective registry analysis, conducted by The European Society for Blood and Marrow Transplantation (EBMT), has reported the relative use of each platform between 2011 and 2015, highlighting a preference for T-cell replete (TCR) transplant with PT-Cy (post-transplant cyclophosphamide) platforms (76% of 2,698 haplo-HSCT), followed by TCR with ATG (anti-thymocyte globulin)-based platforms (21.4%), while *ex-vivo* TCD is only marginally used (16).

**Table 1:** Comparison of current methods for T-cell depletion.

	Protocol name	Biological hypothesis underlying clinical effects	Reported clinical outcomes
	Principle		
**Ex-vivo T-cell depletionTCD platforms**	*CD34^+^ megadose * Positive CD34^+^ selection	Use of G-CSF mobilized PBSC as graft source allows the collection of high numbers of stem cells that are isolated using immune magnetic strategies.	Engraftment 91% Grade II–IV aGVHD 8%/cGVHD 7% TRM 37%–44%
		Slow immune reconstitution leads to high incidence of infection and high relapse rates	
	*Specific alloreactive T-cell depletion* ⇔ Selective CD3^+^/CD19^+^ cell depletion	NK cells, monocytes and dendritic cells are retained, which may contribute to a better immune reconstitution after transplantation	Engraftment 95% Grade II–IV aGVHD 46%/cGVHD 18% 2y TRM 42% 2y relapse rate 31% 2y OS 28%
		Significantly increase of the risk of GVHD compared with positive CD34^+^ selection	
	*Designed graft* ⇔ Specific removal of alloreactive cells	Depletion of TCRαβ^+^/CD19^+^ cells/depletion of CD45RA^+^ naive T cells	Engraftment 97.5% Grade I–II aGVHD 30%/no cGVHD TRM 5% Relapse 24%
		Low incidence of GVHD and NRM, excellent relapse-free survival	
	*Adoptive T-cell add-back*	– Donor-derived Tregs decrease aGVHD	Engraftment 95%, 46 months DFS 56% Grade II–IV aGVHD 15%/no cGVHD
	⇔ Treg/Tcons infusion following haplo-HSCT	– Co-infusion of Treg + conventional T cells fosters immune reconstitution and prevents aGVHD	
		→ Low incidence of GVHD, optimal immune reconstitution, and very low relapse rate	
	*Genetically engineered TK cells add-back*	TK-cell infusions would confer GVL activity and early protective immune reconstitution after haplo-HSCT, while the suicide gene allow the control of GVHD which could be induced by the TK-cells	3y NRM 40% for patients with *de-novo* AML in CR at haplo-HSCT/41% for patients in relapse at haplo-HSCT 3y OS: 49% for patients with *de-novo* leukemias in any CR No GVHD-related deaths or long-term complication (10 of 22 immune reconstituted patients developed aGVHD + 1 patient developed cGVHD)
	⇔ Infusion of donor lymphocytes expressing herpes-simplex thymidine kinase suicide gene (TK-cells) following haplo-HSCT (14)		
**In-vivo T-cell depletion TCR platforms**	*Baltimore protocol*	High-dose PT-Cy	Non-myeloablative conditioning
	T-cell replete (TCR), unmanipulated graft + High doses PT-Cy: 50 mg/kg/day on day +3/+4	– Selectively eliminates the alloreactive donor T cells (mainly naive T cells) without exerting toxic effects on hematopoietic stem cells	Engraftment 85%–90%, OS 40%–45%, Relapse >40%
		↘ Proliferation of alloreactive CD4^+^ effector T cells	Myeloablative regimen
		↘ Survival of alloreactive CD4^+^ and CD8^+^ alloreactive T cells	Decreased relapse compared to NMAC CR1 11%/CR2 26%/active disease 40% Increased OS compared to NMAC CR1 77%/CR2 49%/active disease 38%
		–Preferentially encourages recovery of regulatory T cells	
		→Host regulatory T cells thereby expand shifting the Treg:T-cell ratio in favor of an immunotolerant balance	
	*Beijing protocol*	G-CSF	
	T-cell replete (TCR), unmanipulated and G-CSF primed graft + ATG + Intensive post-graft IS (MTX, CsA, MMF)	Induces T-cell hyporesponsiveness Induces Th2 polarization in BM and PBSC harvests Induces proliferative expansion of regulatory cells, including regulatory T cells, myeloid-derived suppressor cells, and regulatory B cells	Engraftment 99%, Grade II–IV aGVHD 40%/3y cGVHD 50% 3y NRM 17%, 3y relapse 17%, 3y DFS 67%, 3y OS 70%

Adapted from “Evolution of the Role of Haploidentical ´ Stem Cell Transplantation: Past, Present, and Future”, Kwon et al. (7) and “Update on Current Research Into Haploidentical Hematopoietic Stem Cell Transplantation”, Sun et al. (15).

aGVHD, acute GVHD; AML, acute myeloid leukemia; ATG, anti-thymoglobulin; BM, bone marrow; cGVHD, chronic GVHD; CR(#), complete remission (number #); CsA, ciclosporin-A; DFS, disease-free survival; G-CSF, granulocyte colony-stimulating factor; GVL: graft versus leukemia; IS, immunosuppression; MMF, mycophenolate mofetil; MTX, methotrexate; NRM, non-relapse mortality; OS, overall survival; PBSC, peripheral blood stem cell; PFS, progression-free survival; PT-Cy, post-transplant cyclophosphamide; TCD, T-cell depleted; TCR, T-cell replete; TRM, transplant-related mortality; NMAC, non myeloablative conditioning; #y, # years.

Comparing UBC to haploidentical donors in a randomized study, van Besien et al. highlighted that both procedures achieve favorable and similar results for non-relapse mortality, relapse, and survival (17). Concerning the use of 9/10 MMUD, a high cumulative incidence of grade II–IV acute GVHD (aGVHD) was previously reported, which reached 69% in some cohorts (18–20) and restricted the use of such donors. Recent studies introducing PT-Cy instead of historical anti-thymoglobulins (ATG) as GVHD prophylaxis show significantly lower rates of aGVHD using 9/10 MMUD (21, 22). One question being addressed now under clinical trials is whether haploidentical donor or 9/10 MMUD is a better alternative donor (NCT01597778, NCT03250546).

However, practical considerations, such as lower costs, faster availability, and feasibility of donor-lymphocyte infusions, tilt the balance in favor of haplo-HSCT compared with both 9/10 MMUD and UBC (8–10).

### 2.2 Outcomes of Haplo-HSCT in Adult Populations

Considering current standards of care, studies converging toward the beneficial use of haplo-HSCT are increasing as outlined below.

In leukemia, two prospective studies have highlighted that haplo-HSCT was similarly effective as MSD for both acute myeloid leukemia (AML) and acute lymphoid leukemia (ALL) using the Beijing protocol (23, 24). In high-risk leukemia patients, a retrospective study suggested that PT-Cy haplo-HSCT may have a greater graft versus leukemia (GVL) effect, compared with MSD (25). In terms of cGVHD, PT-Cy haplo-HSCT showed better outcome than 8/8 MUD, with similar overall survival (OS) (26). Compared with UBC, in retrospective studies and one randomized study, results differ depending on the strategy used, but PT-Cy haplo-HSCT was as successful as the haplo-cord strategy for OS (17, 27), while being more effective than single-cord transplantation (28, 29).

In ALL with positive residual disease, the haplo-HSCT cohort had a lower 3-year cumulative incidence of relapse (CIR) and higher 3-year probability of leukemia-free survival (LFS) and OS without any difference for non-relapse mortality (NRM) compared with the MSD cohort (30).

In non-Hodgkin lymphoma, retrospective studies have compared the results of PT-Cy haplo-HSCT to single-unit UBC, achieving better results in terms of OS with haplo-HSCT especially when bone marrow is used as the stem cell source (29, 31). In Hodgkin lymphoma, haplo-HSCT compared in large registry studies to both MSD and MUD showed similar survival outcomes compared with MSD and lower cGVHD compared with MUD (32, 33).

Haplo-HSCT has also shown promising results in non-malignant disorders: for example, in sickle cell disease, using both TCD and TCR platforms (34–37); in patients with thalassemia major, using the Baltimore strategy (38); in anaplastic anemia as salvage treatment in patients without an MSD and failing after immunotherapies (39) but also as upfront therapy using the Baltimore protocol (40, 41); and in primary immunodeficiency disorders for patients with high-risk features (42) especially in low-income settings in which family donors are the only available option (43).

Through a meta-analysis of 30 studies and 22,974 recipients (44), PT-Cy haplo-HSCT was associated with increased all-cause mortality compared with MSD but similar all-cause mortality compared with MUD and reduced all-cause mortality compared with MMUD. Considering NRM, PT-Cy haplo-HSCT was associated with worse outcomes compared with MSD but better outcomes compared with MUD and MMUD. In terms of relapse, PT-Cy haplo-HSCT was associated with similar outcome compared with MSD and MMUD but showed increased relapse compared with MUD.

### 2.3 KIR Genotyping as an Insight to Identify the Best Haploidentical Donor

Favorable practical aspects of using a haploidentical donor and accumulation of evidence of improved outcomes achieved with T-cell replete platforms have led to a 291% increase of haplo-HSCT in Europe between 2005 and 2015 according to the 2015 EBMT activity survey report (45). This trend continues since the use of haploidentical donors encountered a 11% increase between 2018 and 2019, according to their last report (46). In particular, 42% of pediatric patients (<18 years old at transplant) undergoing aHSCT with a related donor receive a transplant from a haploidentical relative (46). A comprehensive list of aHSCT indications published in 2019 has specified situations where mismatched alternative donors (MMAD)—i.e., MMUD, UBC, or haploidentical donors—ought to be performed, highlighting the ever wider applications of this procedure (47).

Johns Hopkins cohort data have highlighted that at least 1 and on average 2.7 first-degree relative haploidentical donors would be available for 95% of patients suffering from hematological malignancies. Second-degree relative haploidentical donors have also been successfully used (48–50). The democratization of using haploidentical donors, therefore, leads to a major paradigm shift: while donor availability represented the main drawback for years, the issue now becomes one of finding the best haploidentical donor among several potential ones.

Recent optimization of the resolution/cost ratio for genetic technologies has enabled genomics to become effective in the field of aHSCT, improving HLA typing quality and providing alternative means to assess the compatibility and predict the potential alloreactivity within a donor/recipient (D/R) couple. Natural killer (NK) cell alloreactivity assessment through killer immunoglobulin-like receptor (KIR) genotyping currently represents one of the most promising perspectives.

## 3 Potential of NK Cells in Allogeneic HSCT

### 3.1 NK Cell Education in Autologous Settings

Natural killer cells are innate lymphoid cells involved in early immunity against infectious agents and tumors (51). They work through cytolytic activity and production of cytokines, features regulated by interactions of germline-encoded receptors with their ligands, including major histocompatibility complex (MHC) molecules (52, 53). Two main groups of receptors interact with MHC class I molecules: lectin-like CD94/NKG2 and immunoglobulin superfamily KIR. Here, we focus on KIR as they are highly polymorphic. The cytotoxicity of NK cells is considered as a feature of innate immunity as their receptors are not dependent on somatic rearrangement, although KIRs are also expressed on some T cells. They are regulated by undergoing a process called “education” in humans (“licensing” in murine models) to prevent inappropriate activation of NK cells against normal cells.

An NK cell interacts with an MHC class I molecule *via* one of its cognate receptors to become competent, i.e., to be able to activate itself against abnormal cells (54). NK cells that have not had any contact with an MHC class I molecule remain inactive. Thus, each competent NK cell maintains self-tolerance to autologous healthy cells through an inhibitory receptor, while it can recognize and kill abnormal cells that have downregulated MHC class I molecules, according to the missing-self theory (55).

### 3.2 NK Cell Alloreactivity in aHSCT

As MHC and KIR genes are located on chromosomes 6 and 19, respectively, they segregate independently. In a non-autologous setting, such as aHSCT, donor NK cells can be alloreactive when they express inhibitory KIR that are not engaged by the MHC class I molecules present on the cells of the recipient (52). This ability to sense missing-self ligands is the rationale for an alloreactivity that triggers GVL without promoting GVHD, and is supported by *in-vitro* functional studies (56).

Moreover, the kinetics of recovery and rates of early post-transplant NK cells reached during the period of severe lymphopenia tend to correlate with lower relapse rates and better survival (57, 58) indicating that NK-medicated alloreactivity could be responsible for an early crucial GVL effect.

Considering that i) the current nomenclature of KIR reports 13 genes [*KIR2DL1*, *KIR2DL2/2DL3*, *KIR2DL4*, *KIR2DL5A*, *KIR2DL5B*, *KIR2DS1*, *KIR2DS2*, *KIR2DS3*, *KIR2DS4*, *KIR2DS5*, *KIR3DL1/3DS1*, *KIR3DL2*, and *KIR3DL3*; and 2 pseudogenes: *KIR2DP1* and *KIR3DP1* (59)], ii) genes may either be present or absent on a haplotype, and iii) 1,532 KIR alleles described to date, then KIR/MHC interactions are expected to have massive combinatorial possibilities, shaping NK alloreactivity in aHSCT.

### 3.3 Current Recommendations for Haploidentical Donor Selection and the Place of KIR

Taking conclusions from the literature, in 2019, the EBMT published recommendations for donor selection in haplo-HSCT (**Table 2**) (60). On TCD platforms, “NK alloreactive donor” is ranked as the second criterion of interest, whereas “donor with KIR ligand match for the recipient” is ranked as the seventh criterion among eight on TCR platforms, probably because T-lymphocyte alloreactivity blurs that of NK cells. However, the EBMT does not recommend which predictive strategy should be used to predict NK alloreactivity, while several models exist that can be conceptually opposed, as reviewed below.

**Table 2:** EBMT recommendations for haploidentical donor selection, by order.

T-cell-depleted haploidentical transplants	T-cell-replete haploidentical transplants
No DSAs (MFI < 1,000)	No DSAs (MFI < 1,000)
NK cell alloreactive donor	Younger donor over older donor
Younger donor over older donor	Male donor for a male recipient
Male donor for a male recipient	Sibling or offspring donor over parent donor
First-degree relative over second-degree HLA half-matched donor	Between parent donors, the father is preferred over the mother
Between parent donors, the mother is preferred over the father	ABO matched is preferred to minor ABO mismatch to major ABO mismatched donor^a^
ABO matched donor	Donor with KIR ligand match for a recipient of HHCT^a^
CMV seropositive donor for CMV seropositive recipients First-degree relative over second-degree HLA half-matched donor Donor with NIMA mismatch over NIPA mismatch for a recipient of HHCTa	Donor with NIMA mismatch over NIPA mismatch for a recipient of HHCT^a^

From “The European Society for Blood and Marrow Transplantation (EBMT) consensus recommendations for donor selection in haploidentical hematopoietic cell transplantation” Ciurea et al. (60).

^a^Conclusive data available with the Beijing protocol only.

## 4 KIR Alloreactivity Assessment

### 4.1 Basic Concepts

KIR proteins are characterized by their extracellular immunoglobulin-like domain which determines ligand specificity (61, 62) and by the length of their cytoplasmic tail which determines their function (63). The KIR nomenclature is based on these features: KIR2Dxx and KIR3Dxx have two and three extracellular domains, respectively, while KIRxDSx and KIRxDLx have a short activating or a long inhibitory cytoplasmic tail, respectively. KIR2DL4 is an exception and can be activating or inhibitory depending on allelic polymorphism (64).

Most of the KIR ligands are MHC molecules, as reported in **Table 3**. HLA-C1 and HLA-C2 epitopes refer to the amino acid at position 80 within the α1 domain of HLA class I heavy chain: asparagine residue or lysine residue defines C1 or C2 epitopes, respectively (66). C1 and C2 epitopes are mutually exclusive and can be found in different proportions across populations (67). A similar split can be described for HLA-B molecules between HLA-Bw4 and HLA-Bw6, depending on the amino acid sequence between positions 77 and 83 of α1 domain of the HLA class I heavy chain (68). However, certain HLA-A and HLA-C antigens also bear the Bw4 and Bw6 epitopes, respectively (69).

**Table 3:** Human KIR and their cognate ligands.

Inhibitory KIR		Activating KIR
KIR2DL1	HLA-C2 epitope	KIR2DS1	HLA-C2 epitope
KIR2DL2/3	HLA-C1 epitope	KIR2DS2	Unknown
KIR2DL4	HLA-G	KIR2DS3	Unknown
KIR2DL5A/B	Unknown	KIR2DS4	Subsets of HLA-C and HLA-A*11
KIR3DL1	HLA-A and HLA-B alleles encoding Bw4 epitope	KIR2DS5	HLA-C (variable)
KIR3DL2	HLA-A*03 and HLA-A*11	KIR3DS1	HLA-F
KIR3DL3	Unknown	KIR2DL4	HLA-G

Adapted from Boudreau and Hsu, Natural killer cell in human health and disease (65).

Since NK cell alloreactivity in aHSCT settings can be modulated by KIR/MHC interactions within the D/R couple, KIR and MHC typing can be used to predict NK alloreactivity.

### 4.2 Main Models Predicting NK-Mediated Alloreactivity in aHSCT

#### 4.2.1 Ligand–Ligand Model

**Figure 1:**
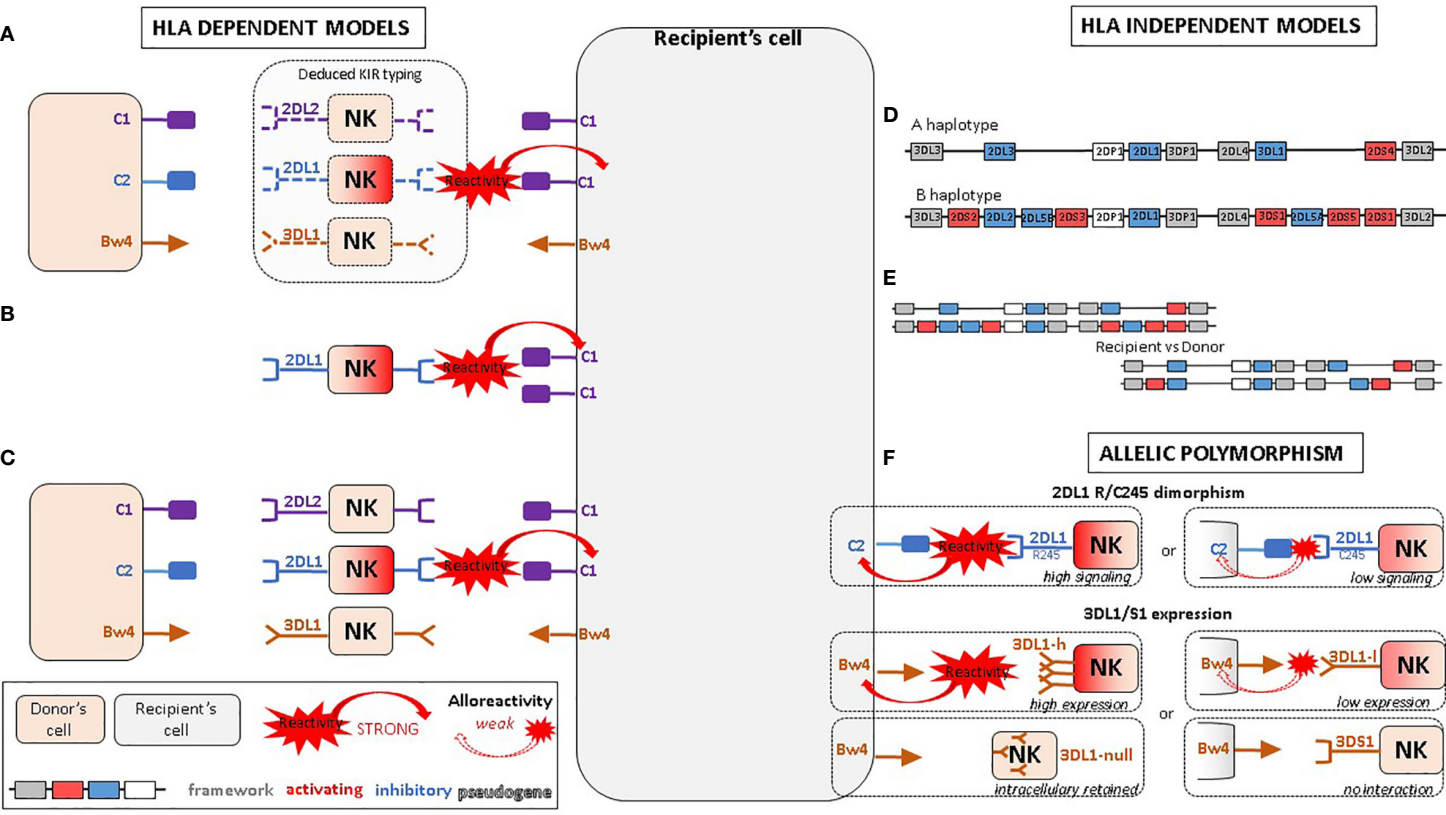
Proposed NK alloreactivity mechanisms in aHSCT according to different models. **(A)** Ligand–ligand model confronts the MHC of the donor with the MHC of the recipient: KIR genotyping is unknown and NK alloreactivity of the donor toward host cells is expected when the recipient lacks MHC class I ligand present in the donor. **(B)** Receptor–ligand model considers the KIR of the donor and the MHC of the recipient: if at least one KIR gene expressed in the donor does not recognize any of the MHC molecules of the recipient (“missing-ligand”), the NK cells of the donor will increase their cytotoxic activity. **(C)** Educational models consider the MHC class I molecules of the donor and recipient and the KIR typing of the donor. It should reflect the “education” process required for NK cells to become competent. **(D)** The KIR haplotypes of the donor: the B/x of the donor and particularly those carrying Cen-B/B are expected to be more alloreactive toward the cell of the recipients, as they carry mostly activating KIR genes. **(E)** KIR matching models represent the number of aKIR and/or iKIR gene present in the donor but absent in the recipient and vice versa. **(F)** KIR polymorphism leads to KIR molecules with relevant biological differences.

First proposed by the Perugia team (70), this model compares the donor and recipient KIR ligands while KIR genotyping is not considered for either of them (**Figure 1A**) . The underlying hypothesis is that the donor NK cells would be alloreactive toward host cells when the recipient is lacking MHC class I ligand present in the donor. As it does not consider whether the cognate KIR is present or not within the donor, this model does not take account of the educational process. The suitable KIR mismatch (GVH direction) is defined as the donor possessing a KIR ligand, an MHC class I epitope, which is absent in the recipient. The IPD database proposes online assessment of mismatches (GVH direction, HVG direction, both or none) by entering the D/R couple HLA-B and HLA-C typing (71). As HLA-A may carry Bw4 or A3/A11 epitopes, this algorithm only approximates ligand–ligand mismatches. Moreover, it does not consider HLA-Bw4 that might be carried by an HLA-C molecule.

Using TCD haplo-HSCT settings, Ruggeri et al. found that alloreactive NK cells in the GVH direction helped to promote engraftment and graft-versus-tumor effect, resulting in reduced risk of leukemia relapse and better survival in adults with AML without increasing the rate of GVHD (56).

#### 4.2.2 Receptor–Ligand/Missing-Ligand Model

Leung et al. proposed an alternative model that considers the donor KIR in relation to the MHC of the recipient, in other words, the KIR receptor of the donor with the KIR ligand of the recipient (72) (**Figure 1B**). Donor MHC typing and recipient KIR typing are not required for this model. The underlying hypothesis is that if at least one KIR gene expressed in the donor does not recognize any of the MHC molecules of the recipient (“missing-ligand”), the NK cell inhibition of the donor will be reduced and consequently their cytotoxic activity will be increased.

The KIR mismatch is defined as the donor possessing inhibitory KIR for which the recipient lacks a ligand. The suitable donor according to this model has at least one KIR receptor–ligand mismatch but should exhibit at least one inhibitory KIR specific for a recipient ligand to maintain NK cell immunoregulatory function, to reduce the risk of NK cell autoimmunity post-transplant, although no case of such autoimmunity has yet been observed (73).

KIR mismatching using the receptor–ligand model shows a trend toward improved overall survival and disease-free survival, as well as decreased risk of relapse (74). In the original study of pediatric patients with high-risk leukemia, given CD34^+^ selected haploidentical graft, Leung et al. found that NK alloreactivity based on this model predicted the risk of leukemia relapse more accurately than the ligand–ligand model (72).

#### 4.2.3 Educational/Missing Licensing Proof Models

Nowak et al. described models that take into account the “education” process required for NK cells to become competent alloreactive NK cells: the presence of a KIR and its cognate MHC molecule in the donor (75) (**Figure 1C**). They, therefore, consider both donor and recipient MHC class I molecules as well as the KIR type of the donor. If the donor possesses both a KIR and its ligand, the NK cells of the donor will be licensed and fully alloreactive. Conversely, if the donor possesses the KIR but not its ligand, the NK cells of the donor will not be licensed. This model has mainly been described for the four main inhibitory KIR genes and their MHC ligands: 2DL1 with C2, 2DL2/3 with C1, 3DL1 with Bw4, and 3DL2 with A3/A11. The KIR mismatch leading to alloreactivity is obtained when the donor has licensed NK cells—i.e., KIR and its cognate MHC ligand—but the recipient lacks the cognate MHC ligand (for example donor 2DL1+ C2+ but recipient C2−).

Counterintuitively, this clinical study (75) has shown that recipients not lacking the KIR–ligand of the cognate donor experienced better overall survival in leukemia patients, in contradiction with the receptor–ligand model (76). Nowak et al. have suggested that the interaction between KIR molecules and their cognate MHC ligands in the recipient contributes to post-transplant immunosurveillance of malignancy, but it could also be argued that the plasticity of education could blur the concept, as NK cell education is a dynamic process during which NK cells can calibrate their functional potential to the MHC ligands present (77). Indeed, the transfer of mature NK cells from one MHC environment to another results in reshaping of the functional potential based on the inhibitory input of the new MHC setting (78).

Nowak et al. also hypothesize that couples predicted as alloreactive by the receptor–ligand model could have lymphocytes remaining largely hyporesponsive to stimulus, as the receptor–ligand model does not take into account donor NK cell education (79). However, the proportion of favorable couples fitting the educational model is small, meaning that only few D/R couples could benefit (76).

This educational model has also been described for the activating KIR and their ligands (80), and especially for KIR2DS1 and its C2 ligand, with donor 2DS1+ C1+ NK cells being fully educated and capable of recognizing their ligands on the C2+ leukemia cells of the recipient. Conversely, donor 2DS1+ C1− NK cells remain hyporesponsive regardless of the ligands exhibited by leukemic cells, highlighting that education differs between activating and inhibitory KIR (81, 82).

#### 4.2.4 Haplotype-Based Models of the Donor

Cooley et al. have defined KIR haplotypes that correlate with the content of activating KIR within a cohort of recipients undergoing aHSCT from unrelated donors (83) (**Figure 1D**). Only donor KIR typing is required for this model. The underlying hypothesis is that certain donors are expected to be more alloreactive toward recipient cells, based on consideration of the activating and inhibitory gene content of KIR haplotypes in the leukocyte receptor complex (LRC) at 19q13.4 (52, 84). The genetic proximity between each KIR gene (2.4 kb) leads to a transmission of haplotypes (85, 86). Haplotypes are split into a centromeric (Cen) and a telomeric (Tel) part delimited by the framework genes *KIR3DL3*/*KIR3DP1* and *KIR2DL4*/*KIR3DL2*, respectively. The gap between *KIR3DP1* and *KIR2DL4* measures 14 kb and represents a hotspot for crossing-over (recombination) between Cen and Tel regions (87).

Thus, Cooley et al. have standardized gene content into three centromeric units (Cen-A, Cen-B1, and Cen-B2) and two telomeric units (Tel-A and Tel-B) (83). The combinations of Cen and Tel units lead to the assessment of the KIR-B content score. KIR-B content score can be easily calculated by entering the KIR typing of the donor by referring to the IPD database (88).

The A-haplotype contains seven genes (*KIR3DL3*, *KIR2DL3*, *KIR2DL1*, *KIR2DL4*, *KIR3DL1*, *KIR2DS4*, *KIR3DL2*) and two pseudogenes (*KIR2DP1* and *KIR3DP1*). As it mainly contains KIRDL genes, the A-haplotype could be considered as predominantly inhibitory. The polymorphism of this haplotype does not depend on gene presence/absence as it has fixed gene content but mainly depends on allelic polymorphism of the inhibitory KIR, which is generally greater than that of the activating KIR (89). B-haplotypes can have up to 12 genes and two pseudogenes, 8 of which are specific to it: *KIR2DS2*, *KIR2DL2*, *KIR2DL5B*, *KIR2DS3*, *KIR3DS1*, *KIR2DL5A*, *KIR2DS5*, and *KIR2DS1*. The B-haplotype is defined by the presence of at least one of these 8 genes even if it contains any gene of haplotype A. Gene content of B-haplotypes is highly variable, and since they contain one or more KIRxDSx genes, they are generally considered to have a more activating profile compared with A.

Thus, all individuals can be categorized as homozygous group A KIR haplotype (A/A) or having at least one group B haplotype (B/x). In their studies, Cooley et al. have shown that significantly reduced relapse was achieved with donors having KIR-B content score ≥2 compared with 0. As the beneficial effects of KIR B/B donors are greater than double those observed when using KIR A/B diplotype donors, A-haplotypes could also have a detrimental impact on aHSCT outcomes. Comparing centromeric and telomeric regions independently, Cen-B and Tel-B motifs both contributed to relapse protection and improved survival, but Cen-B homozygosity had the strongest independent effect on cumulative incidence of relapse, overall survival, and DFS (83).

#### 4.2.5 Gene–Gene/KIR Matching Models

Gene–gene mismatch within the D/R couple—i.e., KIR gene present in the donor but absent in the recipient and vice versa—has been correlated with discrepant clinical outcomes (**Figure 1E**). Symons et al. have shown that haploidentical D/R couples who differed in iKIR gene content had a significantly improved overall survival, event-free survival, and lower relapse rates without any increase of NRM when compared with those patient–donor pairs with identical iKIR gene content (90). This was found for patients with lymphoid diseases as well as myeloid diseases. Conversely, Sahin et al. have shown that KIR matching with an MSD had a protective effect on relapse and occurrence of cGVHD (91). Various scoring strategies can be made from this gene–gene model by assessing the differences between the number of activating and inhibitory KIR genes in the donor and recipient.

### 4.3 Allelic Polymorphisms and NK Alloreactivity in aHSCT

So far, the approaches described above have simply taken into account the presence/absence of KIR genes without considering their polymorphism. A total of 1,532 KIR alleles have been described for KIR genes to date (89) (**Table 4**). This allelic polymorphism leads to KIR molecules with relevant biological differences, such as i) intracellular retention of KIR receptors or low cell-membrane expression (92–94), ii) premature termination codon (null alleles), iii) production of soluble receptors (95), iv) variability in ligand affinity (96), and v) diversity in signal transduction capability (97). Notably, recent clinical studies underline the relevance of *KIR2DL1* (98) and *KIR3DL1* polymorphisms (99) for the selection of aHSCT donor.

**Table 4:** KIR polymorphism, IPD database Release 2.10.0 (December 2020).

Gene	*2DL1*	*2DL2*	*2DL3*	*2DL4*	*2DL5*	*2DS1*	*2DS2*	*2DS3*
Alleles	173	34	64	112	91	33	64	71
Proteins	65	15	35	58	40	12	21	23
Nulls	7	0	1	0	1	0	0	2
**Gene**	***2DS4***	***2DS5***	***3DL1***	***3DS1***	***3DL2***	***3DL3***	***2DP1***	***3DP1***
Alleles	39	88	183	39	165	228	40	108
Proteins	20	38	92	22	115	112	0	0
Nulls	20	0	3	1	1	1	0	0

From the IPD database (https://www.ebi.ac.uk/ipd/kir/stats.html) (89).

#### 4.3.1 2DL1 245 R/C Dimorphism

KIR2DL1 receptors can be split into two groups according to the amino acid at position 245 of its transmembrane domain: KIR2DL1-R245 and KIR2DL1-C245 with arginine or cysteine, respectively (**Figure 1F**). KIR2DL1-R245, known to signal stronger than KIR2DL1-C245 as it can recruit more Src-homology-2 domain-containing protein tyrosine phosphatase 2 and beta-arrestin, shows higher inhibition of lipid raft polarization at the immune synapse and has less downregulation of cell-surface expression on interaction with its ligand (97).

A clinical study from one center, including 313 pediatric patients undergoing aHSCT, showed that recipients who received aHSCT from a KIR2DL1-R245 donor have significantly better survival and lower cumulative incidence of disease progression than those receiving from a KIR2DL1-C245 donor. This effect was similar in patients with AML or ALL and was even higher when patients received a KIR2DL1-R245–positive graft with HLA-C receptor–ligand mismatch in terms of overall survival and compared with those who received a KIR2DL1-C245 homozygous graft (98).

#### 4.3.2 3DL1/S1 Expression

The *KIR3DL1/S1* gene is one of the most polymorphic KIR genes (89) (**Figure 1F**). Allelic versions of this gene are correlated to the expression level and KIR3DL1/3DS1 can be expressed at high (KIR3DL1-h) or low (KIR3DL1-l) cell-surface densities or be retained within the cell (KIR3DL1-n) (100), whereas KIR3DS1 receptors are displayed on the cell surface but do not bind HLA-Bw4 (101, 102). Dimorphism between isoleucine and threonine at position 80 in HLA-Bw4 (Bw4-80I vs. Bw4-80T) is similarly associated with surface expression on healthy cells (103) (80I: high; 80T: low). Allelic combinations of KIR3DL1-h and Bw4-80I are enriched among patients with AML, which suggests that this is a strongly inhibitory combination that may predispose individuals to develop cancer (104). In aHSCT, KIR3DL1/HLA-B combinations with *in-vitro* weak or no inhibition significantly decrease relapse and improve survival compared with strong inhibition combination patients. This effect was greatest in the high-risk group and independent from the benefit of donor activating KIR2DS1 (99).

#### 4.3.3 HLA-B Leader Peptide-21 M/T Dimorphism

KIRs are not the only receptors responsible for NK alloreactivity*. HLA-B* dimorphism at position −21 (methionine or threonine, thus M/T dimorphism) in the segment encoding the leader peptide dictates whether NK cell regulation primarily relies on the KIR or another important heterodimer receptor, NKG2A/CD94. Subjects carrying HLA-B −21M harbor better-educated NKG2A^+^ NK cells and display a superior capacity to degranulate lytic granules against KIR ligand-matched primary leukemic blasts (105).

## 5 Clinical Significance of Predicted NK Alloreactivity

In spite of the detailed analysis undertaken for all of the models described to date, the clinical significance of predicted NK alloreactivity in aHSCT is still unclear. **Supplementary Table 2** aims to associate the models of alloreactivity described above and the clinical settings under which they have been studied. Studies were selected reflecting the current knowledge about KIR, alloreactivity prediction, and aHSCT outcomes, as discussed in the previous sections. Particular attention has been paid to studies on which the EBMT based its recommendations (60).

In addition to the discrepancies due to alloreactivity scoring strategies, this highlights other factors contributing to the inconsistencies described in the literature. First, the studies are mostly retrospective and some of them are considerably dated and span periods up to 20 years. In such a moving field as aHSCT, standards of care have been greatly impacted by medical improvements over such long periods. Additionally, MHC and KIR genotyping has improved, from serological typing to third generation sequencing. Moreover, the heterogeneity of patients that have been studied, especially in terms of ethnicity, pathologies (myeloid vs. lymphoid malignancies), and ages (pediatric vs. adult), further blurs the conclusions regarding NK alloreactivity and aHSCT outcomes. Considering these two last points, the phenotypic changes of leukemic cells related to both factors are responsible for differentiated efficacy of NK cells toward them. As an example, lower expression of ICAM on leukemic cells weakens ICAM/LFA1 interaction, leading to the decrease of NK cell adhesion and cytolytic activity toward them (106).

More importantly, the different graft procedures greatly impact clinical outcomes and therefore lead to inconsistent conclusions. For example, the T-cell depletion in Perugia’s haploidentical transplant protocol is vigorous with stem cell inocula containing an average of 3 × 10^4^ T cells/kg (70), compared with an average of 1.48 × 10^8^ T cells/kg in the Huang study (107). T cells in the allograft might affect NK cell function and KIR expression *in vivo*, as reported in unrelated aHSCT (108), and the beneficial role of NK alloreactivity in transplantation might be inhibited by coadministration of large doses of T cells. The latter could explain the clear positive effect of NK alloreactivity on clinical outcomes when using TCD platforms, while the impact of this alloreactivity remains uncertain when using TCR platforms. On TCR platforms, and using haploidentical grafts, Russo et al. (57) have shown that donor NK cells proliferate immediately after graft infusion but that the level of alloreactive NK cells decreases significantly following PT-Cy administration. Considering the kinetics of NK cell recovery after haplo-HSCT (109), this could be consistent with a suitable GVL effect led by NK cells within a narrow and early but crucial time lapse.

## 6 Discussion

This work aims to report to what extent the NK alloreactivity predicted through KIR genotyping within a D/R couple impacts aHSCT outcomes. The presentation of each model of alloreactivity prediction highlights that every scoring strategy has limitations in assessing NK education required for NK cells to be functional and efficient. Consequently, clinical outcomes differ depending on the model used for alloreactivity prediction. Every scoring strategy has pros and cons that would influence its use (**Table 5**).

**Table 5:** Pros and cons of scoring strategies for NK alloreactivity assessment.

Models	NK cells of the donor alloreactive toward host cells when:	Pros	Cons
Ligand–ligand model	Recipient is lacking an MHC class I ligand that is present in the donor	No KIR typing requiredEasy to use (online algorithm)	Simple approximation of educational modelApproximate estimation of the mismatches if using the IPD database (does not take into account HLA-Bw4 epitopes related to HLA-A and HLA-C or HLA-A3/11 epitopes)
Receptor–ligand model	At least one KIR gene expressed in the donor does not recognize any of the MHC molecules of the recipient	KIR typing only at genic resolution for donors	
Educational models	Donor has educated NK cells—i.e., KIR and its cognate MHC ligand—but the recipient lacks the cognate KIR MHC ligand	KIR typing only at genic resolution for donorsMost comprehensive model for NK alloreactivity	Delicate process that can be overridden in certain conditions, e.g., high inflammation surroundings such as in aHSCT
Haplotypes	Donor has at least one KIR B haplotype	KIR typing only at genic resolution for donorsEasy to use (online algorithm)	
Gene–gene model	KIR gene is present in the donor but absent in the recipient	Easy to use	Far from any biological underlying process
Allelic polymorphisms	A specific D/R interaction is present	Directly targets a functional gene difference	Multitude of models with variable relevance
			Allelic KIR genotyping (time and cost)
			Complex

Solomon et al. (110) have proposed the first integrative model that takes into account two different methods of predicting NK alloreactivity (receptor–ligand model and the combination of Bx-haplotype and KIR2DS2 presence in donors) but also other factors known to impact haplo-HSCT outcomes such as the relationship between donor and recipient (child, parent, sibling) and D/R mismatches for CMV serostatus, HLA-DR and HLA-DP. This integrative scoring strategy manages to stratify the couples for significant differences in DFS of the recipient and cumulative incidence of relapse, especially when disease risk index (DRI) is high, highlighting the improvements that have been made in this field.

NK education is a fine-tuned process, but recent studies have shown that early post-aHSCT uneducated NK cells expressing KIR for non-self HLA are hyperresponsive. This could be due to their inflammatory *in-vivo* environment (radiation, chemotherapy, infections) that might override the educational process. Unlicensed NK cells could be activated regardless of their educational state by lowering their threshold for reactivity (79). Thus, different models based only on receptor–ligand mismatches have been proposed.

This review focused on KIR-related alloreactivity, but many other receptors are known to impact NK functions, such as natural cytotoxicity receptors (NCRs) which play an important role in killing tumor cells of different origins including leukemic cells (111): for example, NKG2D, which is involved in tumor cell recognition by binding specific stress-inducible molecules, MICA/B and ULBPs (112). There is also a report of a monomorphic receptor on NK cells, NKp44, interacting with HLA-DP (113).

Finally, the genotype/phenotype correlation for KIR molecules is not linear and KIR expression depends on i) NK maturation stage (114), ii) education processes and interactions with MHC class I molecules (54), iii) the surrounding area and notably cytokine profile and density (114), and iv) the KIR gene, its allelic version, copy number, and the haplotype that contains it (94).

Even if the KIR-related NK alloreactivity in aHSCT is poorly understood, the strengths of associations in clinical studies are encouraging for its use for i) donor selection in haplo-HSCT, especially when using TCD platforms; ii) donor selection in case of the 9/10 MMUD (47); and iii) adjustments of immunosuppressive therapies in all aHSCT settings, according to the predicted outcome, i.e., to reduce GVHD prophylaxis when graft failure or delayed immune reconstitution is expected, while intensifying it when GVHD is expected. Prospective studies are currently being run on adults and pediatric populations (NCT02450708, NCT02646839 among others).

A better understanding of the different scoring strategies and their underlying processes is therefore crucial to enable the optimal use of KIR genotyping in medical practice, in aHSCT but also in other fields of immunology such as in renal transplantations, as recently described (115). It is worth noting that KIR/MHC interactions have also largely been described in the genetic prediction of malignancies such as AML (104) or neuroblastoma (116) and outcomes of solid tumors (117), pregnancy disorders (118), or viral disease clearance such as HCV/HBV (119, 120) or HIV (121), highlighting all the potential implications and power of this extraordinary system that is slowly revealing its secrets.

## Author Contributions

AD wrote the manuscript. AA, MTR, JoT, and JaT reviewed the manuscript. MP, JJ, and NN-G supported this work by their constructive comments and suggestions along the writing process. All authors contributed to the article and approved the submitted version.

## Funding

JoT and JaT are funded by the European Research Council (ERC) under the European Union’s Horizon 2020 research and innovation program (grant no. 695551). AD received a 1-year funding from l’Agence Régionale de Santé (ARS): contrat année recherche no. 54-2019-1.

## Conflict of Interest

The authors declare that the research was conducted in the absence of any commercial or financial relationships that could be construed as a potential conflict of interest.

## Publisher’s Note

All claims expressed in this article are solely those of the authors and do not necessarily represent those of their affiliated organizations, or those of the publisher, the editors and the reviewers. Any product that may be evaluated in this article, or claim that may be made by its manufacturer, is not guaranteed or endorsed by the publisher.
