# Supplementary material for: KIR in Allogeneic Hematopoietic Stem Cell Transplantation: Need for a Unified Paradigm for Donor Selection
Source: Front Immunol. 2022 Feb 15;13:821533. doi: 10.3389/fimmu.2022.821533 (PMC8886110; doi:10.3389/fimmu.2022.821533)
Supplement: Supplementary file 1 [file Table_1.docx]

Supplementary Material

# Supplementary Tables

# Supplementary Table 1: Comparison of umbilical blood cord derived products and haploidentical donor for aHSCT

Adapted from “Who is the best alternative allotransplant donor”, Gale *et al* (1) and “Alternative donor transplant of benign primary hematologic disorders”, Tolar *et al* (2)

DLI: donor lymphocytes infusion, LFS: leukemia free survival, TNC: total nucleated cells

|  | **Logistics** | | **Clinical outcomes with current protocols** | | | | |
| --- | --- | --- | --- | --- | --- | --- | --- |
| **aHSCT** | **Advantages** | **Disadvantages** | **GF** | **GVHD** | **Infection** | **Relapse** | **LFS** |
| **UBC** | No risk for donor  Low risk of infectious disease transmission of latent viruses  Easy to stock and deliver | TNC / CD34+ 10x lower than adult donors  Single use, no available DLI  High costs  Autologous product as backup | ++++ | + | ++++ | ++++ | ++ |
| **Haplo-identical**  **donor** | Always quickly available  Low costs  High TNC / CD34+ doses  Possible DLI | Less experience  High costs if *ex-vivo* T-cell depletion | ++ | ++++  Reduced if  T-cell depletion | +++ | ++ | ++ |

**REFERENCES**

1. Gale R, Eapen M. Who is the best alternative allotransplant donor? *Bone Marrow Transplant* (2015) **50**:S40–S42. doi: 10.1038/bmt.2015.94

2. Tolar J, Sodani P, Symons H. Alternative donor transplant of benign primary hematologic disorders. *Bone Marrow Transplant* (2015) **50**:619–627. doi: 10.1038/bmt.2015.1
