# Supplementary material for: KIR in Allogeneic Hematopoietic Stem Cell Transplantation: Need for a Unified Paradigm for Donor Selection
Source: Front Immunol. 2022 Feb 15;13:821533. doi: 10.3389/fimmu.2022.821533 (PMC8886110; doi:10.3389/fimmu.2022.821533)
Supplement: Supplementary file 2 [file Table_2.pdf]

Supplementary Table 2: Most striking clinical studies focusing on KIR-mediated NK alloreactivity in aHSCT.

<u>Hypotheses:</u> aKIR: activating KIR, iKIR: inhibitory KIR, CR: complete remission

<u>Population:</u> AML: acute myeloid leukemia, AMLL: acute myelomonocytic leukaemia, ALL: acute lymphoid leukemia, CLL: chronic lymphoid leukemia ,CML: chronic myeloid leukemia, CMML: chronic myelomonocytic leukemia, CR: complete remission, HL: Hodgkin lymphoma, MDS: myelodysplastic syndroms, MM: multiple myeloma, MPN: myeloproliferative neoplasia, NHL: non-Hodgkin lymphoma

<u>Graft's characteristics:</u> ATG: anti-thymoglobulin, BM: bone marrow, CsA: ciclosporin-A, G-CSF: granulocyte colony stimuling factor, mAbs: monoclonal antibodies, MAC: myeloablative conditioning, MTX: methotrexate, MSD: matched sibling donor, MUD: matched related donor, MMUD: matched unrelated donor, NMAC: non-myeloablative conditioning, NMDP: national marrow donor program, PBSC: peripheral blood stem cell, PT-Cy: post-transplant cyclophosphamid, RIC: reduced intensity conditioning, TCD: T-cell deplete, TCR: T-cell replete

<u>HLA and KIR assessments:</u> PCR: polymerase chain reaction, -SBT: sequence based typing, -SSO: sequence specific oligonucleotide, -SSP: sequence specific primer, RT-PCR: reverse transcriptase PCR SNP: single nucleotid polymorphism

<u>Clinical outcomes:</u> #y: # years, DFS: disease free survival, EFS: event free survival, HR: hazard ratio, NRM: non-relapse mortality, OR: odds ratio, OS: overall survival, p: p-value, PFS: progression free survival, TRM: transplant related mortality

Study<br><br>**EFFECT**<br><br>**Hypotheses**	**Population**	**Graft's characteristics**	**Study design**	**HLA and KIR assessments**	**Main results**
**Ligand/ligand model**					
2002 (3)<br><br>**POSITIVE EFFECT**<br><br>**Ligand/ligand model**	Included pairs, n = 92<br><br>*Ages not reported*<br><br>AML, n=57<br>ALL, n=35	Haploidentical donors<br><br><u>Platform</u><br><br>TCD	**Clinical study**<br><br>*Details not reported*<br><br>+<br><br>**biological proof of concept**<br><br>measurement of NK cell alloreactivity by screening of NK clones	**<u>HLA, donors and recipients</u>**<br><br>*Not reported*<br><br>**<u>No KIR typing</u>**	**<u>Clinical results</u>**<br><br>• **<u>Among the whole cohort</u>**<br><br>If absence of ligand-ligand incompatibility vs presence, respectively:<br>- Rejection 15.5% vs 0%<br>- Grade II-IV aGVHD 13.7% vs 0 %<br><br>• **<u>For AML recipients only:</u>**<br><br>- **ligand-ligand incompatibility in the GVHD direction is the only independent predictor of survival**. If absence of ligand-ligand incompatibility vs presence, respectively:<br><br>- 5y EFS 5% vs 60%<br>- 5y probability of relapse 75% vs 0%<br><br>- **absence of ligand-ligand incompatibility in the GVHD direction is the only independent factor for poor outcome** (HR = 0.33)<br><br>• **<u>in ALL: no effect</u>**

					**<u>Biological conclusions ⇔ proof of concept</u>**<br><br>In humans, ligand-ligand model closely correlates with NK clones killing recipient's targets.<br><br>In murine model, alloreactive NK cells increase engraftment, graft-versus-tumor effect and survival while decrease relapse and prevent GVHD by elimination of recipient APCs
2007 (4)<br><br>**NEGATIVE EFFECT**<br><br>**Ligand-ligand model**	Included pairs, n=116<br><br>*Ages not reported*<br><br>AML, n=34<br>MDS, n=5<br><br>ALL, n=40<br>CML, n=35<br>AMLL, n=2	Haploidentical donors<br><br><u>Platform</u><br><br>TCR with ATG<br><br><u>Graft source</u><br><br>PBSC with G-CSF mobilized BM<br><br><u>Conditioning regimen</u><br><br>Only MAC	Retrospective<br><br>Between November 2002 and October 2005<br><br>Monocentric, Peking University Institute of Hematology (China)	**<u>HLA, recipients and donors</u>**<br><br>HLA-A, -B, -C and -DRB1 at allele-level molecular typing<br><br>**<u>No KIR typing</u>**	**Ligand-ligand mismatch:**<br><br>• **Considering aGVHD**<br><br>- **independent risk factor for aGVHD** (HR=2.48, p=0.01)<br><br>- **increase of aGVHD incidence in the standard-risk group** (87.5 vs 34.3%, p=0.001)<br><br>compared to patients without ligand-ligand mismatch<br><br>• **Considering TRM, OS and relapse**<br><br>- **independent risk factor for OS** (HR=2.23, p=0.049) and **relapse** (HR=4.77, p= 0.017)<br><br>- **higher cumulative relapse rate** (27.1 vs 0%, p=0.007 for AML / 53.7 vs 6.7%, p=0.003 for ALL)<br><br>- **inferior OS rate** (50 vs 81.9%, p=0.040 for AML / 35 vs 74.8%, p=0.044 for ALL)

2018 (5)<br><br>**POSITIVE EFFECT**<br><br>**Ligand-ligand model**<br><br>*Focused on effectiveness considering disease status (remission or not)*	Included pairs, n=144<br><br>Adults only<br><br><u>Myeloid (n=51)</u><br><br>AML, n=32<br>MDS, n=15<br>MPN, n=4<br><br><u>Lymphoid (n=93)</u><br><br>NHL, n=38<br>HL, n=34<br>MM, n=9<br>ALL, n=7<br>CLL, n=5<br><br><u>Remission</u><br><br>CR, n=81<br>No CR, n=63	Haploidentical donor<br><br><u>Platform</u><br><br>TCR with PT-Cy<br><br><u>Graft sources</u><br><br>BM, n= 53<br><br>PBSC, n= 91<br><br><u>Conditioning regimen</u><br><br>MAC, n=19<br><br>RIC, n=31<br><br>NMAC, n=94	Retrospective<br><br>Between December 2009 and December 2014<br><br><u>2 centers:</u><br><br>Institut Paoli Calmettes, Marseille, France<br><br>Humanitas Cancer Center, Rozzano, Italy	<u>**HLA, donors and recipients**</u><br><br>DNA high-level typing<br><br><u>**No KIR typing**</u>	• <u>**If absence of CR**</u><br><br>**Ligand-ligand mismatch correlates with**<br><br>**- Lower 2y relapse incidence**: 18% vs. 42%, p=0.068 (multivariate analysis: HR= 0.21, p=0.013)<br><br>**- Better PFS**: 50% vs. 21%, p = 0.037 (multivariate analysis: HR = 0.42, p = 0.028)<br><br>**- Trend for improved OS**: 50% vs 28%, p=0.141<br><br>when compared to patients without ligand-ligand mismatch, respectively.<br><br>**Same rates of aGVHD** (18% vs. 17%, p = 0.892) **and cGVHD** (18% vs. 7%, p = 0.197) irrespective of the ligand-ligand mismatch.<br><br>• <u>**If recipient in CR**</u><br><br>No significant effect of ligand-ligand mismatch
2019 (6)	Included pairs, n=444	Haploidentical donor<br><br><u>Platform</u>	Retrospective	<u>**HLA, donors and recipients**</u>	**Ligand-ligand mismatch**<br><br>when compared with no ligand-ligand mismatch

**NEGATIVE EFFECT**<br><br>**Ligand-ligand model**<br><br>+ "Host missing ligands", irrespective of the expression in donor. = missing ligand theory → no correlation with transplantation outcomes → is not discussed in the paper	Adults only<br><br><u>Acute leukemia only</u><br><br>AML, n=327<br>ALL, n=117<br><br><u>Remission status</u><br><br>CR1 = 39%<br>CR2= 26%<br>No CR = 35%	TCR with PT-Cy<br><br><u>Graft sources</u><br><br>BM (54%)<br>PBSC (46%)<br><br><u>Conditioning regimen</u><br><br>MAC (54%)<br>RIC (46%)	Between 2009 and 2015<br><br>Multicentric<br><br>Acute Leukemia Working Party of the EBMT, 500 centers worldwide	DNA high-resolution typing of class I and II HLA antigens<br><br>**<u>No KIR typing</u>**	**- decreases 2y OS :** 46.8% vs 53.1%, p=0.11 (multivariate analysis: HR 1.4, p=0.03)<br><br>**- strives for higher relapse:** HR 1.36, p=0.09, especially in patients with AML (HR 1.48, p=0.07)<br><br>**Those effects on OS and relapse are stronger**<br><br>- when using PBSC (compared to BM)<br>- for AML recipients (compared to ALL)<br><br>**No effect on aGVHD, cGVHD, engraftment or NRM**
**Receptor/ligand model**					
2005 (7)<br><br>**POSITIVE EFFECT**<br><br>**Receptor-ligand model**	Included pairs, n=178<br><br>Paediatric + adult<br><br><u>Myeloid (n=133)</u><br><br>AML, n=57	Matched sibling donor<br><br><u>Platform</u><br><br>*Ex-vivo* TCD (mAbs)<br><br><u>Graft source</u>	Retrospective<br><br>Between 1981 and 1998<br><br>Single-center study in New-York, USA	**<u>HLA, donors and recipients</u>**<br><br>Class I and II intermediate resolution (serology, PCR-SSP, or -SSO), and high resolution (-SBT) if needed to confirm HLA identity<br><br>**<u>HLA, only for recipients</u>**	**<u>Missing ligand effect</u>**<br><br>• <u>In AML and MDS</u><br><br>Compared with patients exhibiting all class I ligands for donor KIR, missing ligand effect:<br><br>**- increases DFS** (HR=0.53, p=0.014)<br>**- increases OS** (HR=0.53, p=0.03)<br>**- lower incidence of relapse** (HR=0.41, p=0.04), withstanding multivariate analysis

Recipients lacks HLA ligand for at least one donor inhibitory KIR	CML, n=61<br><br>MDS, n=15<br><br><u>Lymphoid</u><br><br>ALL, n=45	Bone-marrow derived graft<br><br><u>Conditioning regimen</u><br><br>Myeloablative conditioning		high-resolution for HLA-B and -C typing (alleles identification for epitope segregation)<br><br>**<u>KIR, only for donors</u>**<br><br>Gene detection of *KIR2DL1, KIR2DL2, KIR3DL3*, and *KIR3DL1* using PCR SSP	**If lacking 2 HLA ligands** for donor-inhibitory KIR, **even more higher DFS** (p=0.002) **and OS** (p=0.003).<br><br>• **<u>In CML and ALL:</u>**<br><br>No effect on DFS, OS or relapse<br><br>Same risk for GVHD regardless the missing-ligand effect in any disease group
**Educational model**					
2014 (8)<br><br>**NEGATIVE EFFECT**<br><br>**Educational models based on iKIR**	Included pairs, n=283<br><br>Paediatric + adult<br><br>AML, n=133<br>ALL, n=69<br>AL not specified, n=6	<u>Unrelated donors:</u><br><br>10/10, n=193<br><br>9/10, n=72<br><br>8/10, n=17<br><br>7/10, n=1<br><br><u>Platform</u><br><br>TCD, n=48	Retrospective<br><br>Between 2002 and 2010<br><br>Multicentric study in Poland	**<u>HLA, donors and recipients</u>**<br><br>Provided by each transplant center<br><br>**<u>KIR, only for donors</u>**<br><br>Gene detection of all KIR genes using PCR-SSP	**Comparing to recipients possessing HLA ligand cognate with the donor's NK cell licensing system, recipients lacking at least one HLA ligand have**<br><br>- **decreased 4y OS** (death events 83.3% vs. 39.8%, p=0.001, HR=2.97, p=0.001)<br><br>- **decreased 4y PFS** (91.6% vs. 47.7%, p=0.0001, HR=3.45, p=0.0001)<br><br>- **decreased time to progression** (30.0% vs. 17.3%, p=0.013; HR=4.46, p=0.013)

	CL not specified, n=59<br>Lymphoma, n=9<br>MM, n=7	TCR, n=235<br><br><u>Graft sources</u><br><br>BM, n=71<br><br>PBSC, n=201<br><br><u>Conditioning regimen</u><br><br>MAC, n=174<br><br>RIC, n=96			**Those effects are not associated with aGVHD and independent from HLA-mismatch**<br><br>Incidence of aGvHD comparable regardless of educational status groups (66.7% vs. 53.0%, OR=0.94, p=0.36)
		**Donor's haplotype based model**			
2010 (9)<br><br>**POSITIVE EFFECT**<br><br>**KIR-B content score**	Included pairs, n=1409<br><br>Paediatric + adult<br><br>AML, n=1086<br><br>ALL, n=323	<u>Unrelated donors:</u><br><br>10/10, n=687<br><br>9/10, n=361<br><br>8/10, n=213<br><br>Less than 8/10, n=148<br><br><u>Platform</u>	Retrospective<br><br>Transplants facilitated by NMDP between 1988 and 2006<br><br>Multicentric study in USA	**<u>HLA, donors and recipients</u>**<br><br>High-resolution HLA-A, -B, -C, -DRB1, and -DQB1<br><br>**<u>KIR, only for donors</u>**<br><br>Gene detection of all genes using SNP based *KIR*/MALDI-	**<u>In AML, significant protective effect on relapse</u>**<br><br>**- of donor *B/x* vs A/A genotype** (RR=0.72, p=0.003)<br><br>**- of donor Cen-B/B vs Cen-A/A** (RR=0.34, p<.001)<br><br>**- of donor with KIR B–content≥ 2 compared to < 2**<br>- if HLA matched (RR=0.52, p<0.001)<br>- and if HLA mismatched (RR=0.52, p<0.001)<br><br>Same protective trends for donor Tel-B/B vs Tel-A/A (RR=0.52, p<0.07)

Scoring strategy reflecting the aKIR gene content		TCR<br><br>Graft source<br><br>BM, n=942<br><br>PBSC, n=467<br><br>Conditioning regimen<br><br>Myeloablative conditioning		TOF + KIR-B content score	**ALL: no effect**
**KIR matching model**					
2010 (10)<br><br>**POSITIVE EFFECT**<br><br>**Inhibitory KIR gene mismatch**	Included pairs, n=86<br><br>Paediatric + adult<br><br>AML, n=25<br><br>ALL, n=7<br><br>MDS, n=8	Haploidentical donors<br><br>Platform<br><br>TCR<br><br>Graft source<br><br>Bone-marrow derived graft	Retrospective<br><br>Consecutive inclusion from 2 clinical trials, between 1999 and 2007<br><br>Single-center study in Baltimore, USA	**HLA for recipients:**<br><br>PCR-SSOP + PCR-SBT<br><br>**HLA for donors:**<br><br>HLA-A at intermediate resolution	**Compared to recipients from donors with identical KIR gene content, recipients with inhibitory KIR (iKIR) gene-mismatched have**<br><br>- **increased OS** (HR=0.37, p=0.0003)<br><br>- **significant for lymphoid diseases** (HR=0.44, p=.03)<br>- **as well as myeloid diseases** (HR=0.32, p=.004)<br><br>- **improved EFS** (HR=0.51, p=0.01)<br><br>- **lower relapse rate** (cause specific hazard ratio, SDHR=0.53, p=0.025).

	CML/CMML, n=11<br><br>CLL, n=8<br><br>Lymphoma, n=21<br><br>MM, n=6<br><br>High risk malignancies only	Conditioning regimen<br><br>NMAC + PT-Cy		+ HLA-B, -C, -DRB1 and -DQB1 alleles at a high-resolution<br><br>**KIR, donors and recipients**<br><br>Gene detection of all KIR genes using PCR-SSP. Inheritance of B haplotype deter-mined by the presence of specific aKIR and iKIR.	No significant difference in aGVHD, cGVHD, NRM or engraftment failure
**Polymorphism**					
2017 (11)<br><br>**POSITIVE EFFECT**<br><br>**KIR3DL1 level of expression:**<br><br>Strength of 3DL1/Bw4 interaction correlates	Included pairs, n=1328<br><br>Paediatric + adult<br><br>Only AML	Unrelated donor<br><br>10/10, n=716<br><br>9/10, n= 612<br><br>Platform<br><br>TCD, n=112<br><br>Graft sources	Retrospective<br><br>Between 1989 and 2008<br><br>Transplants facilited by NMDP	**HLA, donors and recipients**<br><br>Provided by the Center for International Blood and Marrow Transplant Research<br><br>**KIR, for donors only**<br><br>* Gene detection for all KIR using PCR-SSO or –SSP	**KIR3DL1/HLA-B combinations with *in-vitro* weak or no inhibition**<br><br>when compared to strong inhibition combinations, have<br><br>- **lower relapse** (HR=0.72, p=0.004)<br><br>- **lower overall mortality** (HR=0.84, p=0.03)<br><br>**This effect is**

with NK alloreactivity<br><br>**+ biological proof of concept**<br><br>*In-vitro* testing of NK-cytotoxicity		BM, n=722<br>PBSC, n= 606<br><br>Conditioning regimen<br><br>MAC, n=1123<br><br>RIC, n=186		* PCR-SBT or multiplex to classify *KIR3DL1* as *KIR3DS1,* high, low or null subtypes	- **greater in high-risk group** (relapse: HR=0.54, p<0.001 / mortality: HR=0.74, p<0.008).<br><br>- **independent from the benefit of donor activating KIR2DS1**<br><br>**Biological conclusions:** Correlation between predicted alloreactivity and alloreactivity measurements
2013 (12)<br><br>**POSITIVE EFFECT**<br><br>**KIR2DL1 dimorphism**<br><br>245C or 245R<br><br>245R is a more effective receptor than 245C	Included pairs, n=313<br><br>Paediatric only<br><br>Hematologic malignancies, n=231<br><br>Lymphoid, n=116<br>Myeloid, n=115<br><br>Solid tumors, n=25<br><br>Nonmalignant diseases,<br><br>n=57	MSD, n=86<br><br>MUD, n=98<br><br>Haploidentical donor, n=129<br><br>Platform<br><br>TCD = 154<br><br>TCR = 159<br><br>Conditioning regimen<br><br>MAC = 240<br><br>NMAC = 73	Retrospective<br><br>Between January 2000 and January 2010.<br><br>Monocentric, St Jude Children's Research (USA)	**HLA, donors and recipients**<br><br>HLA-A, -B, -C, and -DRB1 using DNA methods<br><br>**KIR, for donors only**<br><br>Gene detection by PCR-SSP<br><br>*KIR2DL1* functional allele typing using SNP assay	**KIR2DL1-R245 donor compared to 2DL1-C245 donor lead to**<br><br>• **increase survival**<br><br>**- Risk of death if RR donor compared with CC donor** : HR=0.4, p=0.0001<br><br>**- Risk of death if RC donor compared with CC donor**: HR, 0.42, p=0.0013<br><br>This effect :<br><br>- withstands the multivariate analysis<br><br>- is similar for patients with AML or ALL / in the subset of sibling, unrelated, or haploidentical donor / for T-cell depleted or replete grafts / in myeloablative or non-myeloablative settings as well<br><br>- is higher when patients receive a 2DL1-R245+ positive graft with HLA-C receptor-ligand mismatch

					• **higher PFS**<br><br>- **RR donor compared with CC donor**: HR=0.42, p=0.0003<br>- **RC donor compared with CC donor**: HR=0.48, p=.0075<br><br>This effect:<br><br>- withstands the multivariate analysis<br>- is similar in patients with AML or ALL<br><br>**No significant correlation with grade II-IV aGVHD**
**Others**					
2004 (13)<br><br>Model predicting alloreactivity if donor has aKIR and recipient lacks the ligand for its inhibitory counterpart	Included pairs, n=220<br><br>Paediatric + adults<br><br><u>Myeloid (n=112)</u><br><br>AML n= 52, CML n=49, MDS n=11	Matched sibling donor<br><br><u>Conditioning regimen</u><br><br>NMAC = 58<br><br>MAC = 162	Retrospective<br><br>Between January 1994 and April 2002<br><br>**2 centers in Birmingham, UK :** Children's Hospital and	**<u>HLA, donors and recipients</u>**<br><br>- Class I: PCR-SSP + HLA-C with the adequate level to determine C1 or C2 groups<br><br>- *Class II: not mentioned*	**<u>Results in myeloid malignancies</u>**<br><br>1/ Homozygous C2 recipients have decreased 4y OS compared to those carrying at least one C1 allele (31.6% vs 56.1%, p<0.005)<br><br>2/ In the subgroup of recipient C2-homozygoty:<br><br>- the presence of *KIR2DS2* is significantly associated with decreased OS

Focused on association<br><br>C1- recipients with 2DS2+ donors<br><br>**NEGATIVE EFFECT**	<u>Lymphoid (n=108)</u><br><br>ALL n=54, NHL n=43, CLL n=11		Queen Elizabeth Hospital	**<u>KIR, only for donors</u>**<br><br>Gene detection of all genes using PCR-SSP	- but if *KIR2DS2* is absent, OS is not significantly different from those recipients who possess C1 alleles<br><br>3/ No other significant difference between any of the other pairs analysed.<br><br>4/ No significant difference in the rates of aGVHD > grade II in any model<br><br>**<u>No statistical results in lymphoid malignancies</u>**
2005 (14)<br><br>**Descriptive statistics**<br><br>Compare "relapse" group to "non-relapse" group and assess differences in KIR typing ⇔ Correlations between recipients' clinical outcomes and	Included pairs, n=65<br><br>Paediatric + adult<br><br>AML, n= 22<br><br>ALL, n=16<br><br>CML, n= 27	Matched sibling donor<br><br><u>Platform</u><br><br>- TCR, n=31<br><br>- TCD, n=34<br><br><u>Graft sources</u><br><br>BM = 47<br><br>PBSC = 18	Retrospective<br><br>Between 1991 and 2002<br><br>Single center study in Brussel (academisch ziekenhuis–Vrije Universiteit Brussel)	**<u>HLA, donors and recipients</u>**<br><br>molecular techniques for HLA-A, -B, -C, -DRB1, -DQB1 and -DPB1<br><br>**<u>KIR, donors and recipients</u>**<br><br>Gene detection of *KIR2DL1-3, KIR3DL1-2, KIR2DS1-5* and *KIR3DS1* using PCR-SSP	**<u>General statistics</u>**<br><br>- iKIR genes present in most of the donors.<br><br>- Most variations between 2 groups observed in the number of aKIR<br><br>- Higher frequencies of donor aKIR genes in the non-relapsing group compared to the relapsing group but no significant difference in the frequencies of individual KIR genes<br><br>- No significant correlation between donors' total number of aKIR and relapse<br><br>**<u>Donors 2DS1+/2DS2+</u>**

recipients' and donors' KIR typing<br><br>**POSITIVE EFFECT**		<u>Conditioning regimen</u><br><br>Myeloablative conditioning			- **decrease relapse rate** compared other groups: OR=0.18, p=0.03. Effect withstanding the multivariate analysis.<br><br>- **tend to increase 5y OS:** 59.2 and 35.5% in the 2DS1+2DS2+ and other donors, respectively, p=0.109)<br><br>**<u>No statistical association between:</u>**<br><br>- Relapse and recipient *HLA-C* groups only or in combination with the aKIR<br><br>- Presence of a particular KIR gene or association and TRM, aGVHD or cGVHD
**Models comparisons**					
2004 (15)<br><br>**Compare 3 models of alloreactivity prediction**<br><br>- ligand-ligand model	Included pairs, n=36<br><br>Paediatric patients (<18 yo)<br><br>Myeloid malignancy, n=17	Haploidentical<br><br><u>Platform</u><br><br>TCD<br><br><u>Graft source</u><br><br>Graft purification for CD34+ using mAbs	Retrospective<br><br>**2 centers :**<br><br>Memphis (USA)<br><br>Tuebingen (Germany)	**<u>HLA, donors and recipients</u>**<br><br>* Serology for HLA-A, -B, -DR specificities<br><br>* Molecular biology for DRB1: PCR-SSO and -SSP<br><br>* if serologically difficult to split and for oldest samples +	**<u>In order of efficience for relapse of primary disease prediction</u>**<br><br>1) Receptor-ligand model HR=5.3, p=0.0078<br>2) Ligand ligand model HR=2.1, p=0.47<br>3)Cytotoxicicty model HR=1.4, p=0.76<br><br>**<u>1) Receptor-ligand model</u>**<br><br>**Absence of mismatch is associated to high risk of relapse in AML and ALL**

- receptor-ligand model<br><br>- "cytotoxicity model": NK cell cytotoxicity against K562 cells lower than the median 1mo after transplantation should be predictive for high risk of relapse<br><br>**UNDETERMINED EFFECT**	Lymphoid malignancy, n=19	No GVHD prophylaxis (all grafts contain less than 3.10^4 CD3 cells/kg)		PCR-SSP for class I of unrelated donors<br><br>**<u>KIR, donors and recipients</u>**<br><br>Surface expression of KIR molecules using flow cytometry and RT-PCR if KIR expression was difficult to define<br><br>KIR genotyping : PCR-SSP	**<u>2) Ligand–ligand model</u>**<br><br>**-** absence of mismatch is associated to high risk of relapse for AML only<br>- the model misses some high-risk in AML<br>- fails to classify ALL<br><br>**<u>3)"Cytotoxicity model"</u>**<br><br>The worst model in this serie
2010 (10)<br><br>**Compare 3 models of alloreactivity prediction**<br><br>- Ligand-ligand model<br><br>- Haplotypes	Included pairs, n=86<br><br>AML, n=25<br><br>ALL, n=7<br><br>MDS, n=8<br><br>CML/CMML, n=11<br><br>CLL, n=8<br><br>Lymphoma, n=21	Haploidentical donors<br><br><u>Platform</u><br><br>TCR<br><br><u>Graft source</u><br><br>Bone-marrow derived graft	Retrospective<br><br>Consecutive inclusion from 2 clinical trials, between 1999 and 2007<br><br>Single-center study in Baltimore, USA	**<u>HLA for recipients:</u>**<br><br>PCR-SSOP + PCR-SBT<br><br>**<u>HLA for donors:</u>**<br><br>HLA-A at intermediate resolution<br><br>+ HLA-B, -C, -DRB1 and -DQB1 alleles at a high-resolution<br><br>**<u>KIR, donors and recipients</u>**	**<u>iKIR mismatch</u>**<br><br>**Compared to recipients from donors with identical KIR gene content, recipients of inhibitory KIR (iKIR) gene-mismatched have**<br><br>- **improved OS** (HR=0.37, p=0.0003)<br><br>- **significant for lymphoid diseases** (HR=0.44, p=0.03)<br>- **as well as myeloid diseases** (HR=0.32, p=0.004)<br><br>- **improved EFS** (HR=0.51, p=0.01)<br><br>- **lower relapse rate** (cause specific hazard ratio, SDHR=0.53, p=0.025).

- Gene-gene model for aKIR and iKIR<br><br>**POSITIVE EFFECT**	MM, n=6<br><br>High risk malignancies only	<u>Conditioning regimen</u><br><br>NMAC + PT-Cy		Gene detection of all KIR using PCR-SSP. Inheritance of B haplotype determined by the presence of specific aKIR and iKIR.	No significant difference in aGVHD, cGVHD, NRM, or engraftment<br><br>**<u>Haplotype mismatch</u>**<br><br>**AA recipient transplanted with Bx donor compared to AA donor have:**<br><br>- **improved OS** (HR=0.30, p=0.01)<br><br>- **improved EFS** (HR=0.47, p=0.05)<br><br>- **lower NRM** (HR=0.13, p=0.046)<br><br>No significant effect on relapse, engraftment failure, aGVHD or cGVHD<br><br>No significant correlation when recipient is Bx<br><br>**<u>No significant difference with other models</u>**
2014 (16)<br><br>**Compare 2 models of alloreactivity prediction**	Included pairs, n=57	Haploidentical donor<br><br><u>Platform</u>	Prospective<br><br>Between 2004 and 2009	**<u>HLA, donors and recipients</u>**<br><br>already known	**<u>KIR haplotypes</u>**, **<u>regardless of the pathology</u>**<br><br>**decrease relapse for recipients of Bx donors compared to AA donors** (p=0.001).<br><br>This effect

- ligand-ligand model<br><br>- KIR haplotypes (⇔ KIR-B content score)<br><br>**UNDETERMINED EFFECT**	Adults only, refractory diseases<br><br>AML,n=36<br>ALL,n=8<br><br>NHL/ mantle cell lymphoma/ CLL, n=6<br><br>CML/ CMML/ SMD,n=3<br><br>MM, n=4<br><br><u>Remission status</u><br><br>CR, n=29<br><br>PR, n=28	TCD (ex-vivo CD3/CD19 depletion)<br><br><u>Conditioning regimen</u><br><br>Reduced intensity conditioning	Multicenter phase I/II study, 7 centers in Germany	<u>**KIR, for donors only**</u><br><br>* Gene detection of all KIR genes using real time-PCR<br><br>* Quality insurance through commercial typing kits	**- is greater if recipient is in partial remission** (p=0.008) compared to CR (p=0.297)<br><br>**- is greater in AML recipients compared to ALL** recipients<br><br>No effect on reconstitution of NK cells, no effect on NRM<br><br><u>**Ligand-ligand mismatch, for AML recipients only**</u><br><br>- reduces EFS compared to KIR matched pairs16.0 % vs 53.0 % respectively, HR=2.27, p=0.045
2017 (17)<br><br>**Compare 3 models of alloreactivity prediction in**	Included pairs, n=106<br><br>Paediatric + adult	MSD, n=36<br><br>MUD, n=22<br><br>MMUD, n=35<br><br>Unknown, n=13	Retrospective<br><br>Monocentric, Niigata University	<u>**HLA, recipients and donors**</u><br><br>Serologic typing at of HLA-A, -B, and -DRB1 until 2006 and by DNA typing of	<u>**Donor Bx haplotype compared to AA haplotype**</u><br><br>**- increases risk for grade III to IV aGVHD**<br>A/A: 4.9% vs B/x: 20.0%; p= .02<br><br>**- especially if associated with receptor-ligand mismatch**

**Japanese population**<br><br>Ligand-ligand model<br><br>Receptor-ligand model<br><br>Haplotype based models<br><br>**NEGATIVE EFFECT**	AML, n=44<br><br>ALL, n=28<br><br>CML, n=14<br><br>MDS, n=9<br><br>NHL, n=11	Platform<br><br>TCR without ATG<br><br>Graft sources<br><br>BM, n=86<br><br>PBSC, n=20<br><br>Conditioning regimen<br><br>MAC, n=90<br><br>RIC, n=16	Medical Hospital (Japan)<br><br>Between January 1989 and September 2011	HLA-A, -B, -C, and -DRB1 from 2007<br><br>HLA allele data were retrospectively retyped if possible<br><br>**KIR, for donors only**<br><br>Gene detection of all genes using PCR-SSO	no missing ligand: 7.7% vs 1 missing ligand and A/A: 5.3%, vs 1 missing ligand and B/x: 25.0; p=.047<br><br>**No difference in 5-year OS, relapse and NRM**
2018 (18)<br><br>**Compare several models of alloreactivity prediction**<br><br>- Haplotypes<br><br>- Ligand-receptor	Included pairs, n=208<br><br>Adults only<br><br>ALL, n=36<br><br>AML, n=71	Haploidentical donor<br><br>Platform<br><br>TCR platform with PT-Cy<br><br>Graft sources	Retrospective<br><br>Between October 2005 and December 2016<br><br>Single institution	**HLA, donors and recipients**<br><br>High-resolution HLA-A, -B, -C, -DRB1, -DQB1, and -DPB1<br><br>using Sanger sequencing of at least exon 2 and 3 of class I loci and at least exon 2 of class II loci	**KIR receptor-ligand mismatch for iKIR**<br><br>- **improves OS** (HR=0.63; p=0.050)<br>- **improves DFS** (HR=0.57; p=0.012)<br>- **decreases relapse/progression** (HR=0.41;p=0.001)<br><br>**When compared to donors with A/A haplotypes, donors KIR B/x with 2DS2**<br><br>- **improve OS** (HR=0.43; p=0.005)<br>- **improve DFS** (HR=0.45; p=0.003)

- Ligand–ligand<br><br>- KIR B content score<br><br>- aKIR educational models<br><br>- Effect of specific aKIR: *KIR2DS1* and *KIR2DS2*<br><br>**POSITIVE EFFECT**	MDS/MPN/CML, n=42<br><br>NHL/HL/CLL, n=51<br><br>MM, n=5<br><br>Others, n=3	PBSC, n=137<br><br>BM, n=71<br><br><u>Conditioning regimen</u><br><br>MAC, n=86<br><br>NMAC, n=122		**<u>KIR, donors and recipients</u>**<br><br>Gene detection of all genes using PCR-SSP	- **decrease relapse/progression** (versus B/x without 2DS2 : HR= 0.43, p=0.024 / versus A/A haplotype: HR=0.58, p=0.187)<br>- increase NRM (for A/A versus B/x with 2DS2: HR=5.74, p=0.001 / for B/x without 2DS2 versus B/x with 2DS2: HR=3.76, p=0.039)<br><br>**No correlation for the other predictive models**<br><br>**→ Design of an algorithm for donor selection taking NK alloreactivity into account**

<u>STUDIES</u>

1. Gale R, Eapen M. Who is the best alternative allotransplant donor? *Bone Marrow Transplant* (2015) 50:S40–S42. doi:10.1038/bmt.2015.94

2. Tolar J, Sodani P, Symons H. Alternative donor transplant of benign primary hematologic disorders. *Bone Marrow Transplant* (2015) 50:619–627. doi:10.1038/bmt.2015.1

3. Ruggeri L, Capanni M, Urbani E, Perruccio K, Shlomchik WD, Tosti A, Posati S, Rogaia D, Frassoni F, Aversa F, et al. Effectiveness of donor natural killer cell alloreactivity in mismatched hematopoietic transplants. *Science* (2002) 295:2097–2100. doi:10.1126/science.1068440

4. Huang X-J, Zhao X-Y, Liu D-H, Liu K-Y, Xu L-P. Deleterious effects of KIR ligand incompatibility on clinical outcomes in haploidentical hematopoietic stem cell transplantation without in vitro T-cell depletion. *Leukemia* (2007) 21:848–851. doi:10.1038/sj.leu.2404566

5. Wanquet A, Bramanti S, Harbi S, Fürst S, Legrand F, Faucher C, Granata A, Calmels B, Lemarie C, Picard C, et al. Killer Cell Immunoglobulin-Like Receptor-Ligand Mismatch in Donor versus Recipient Direction Provides Better Graft-versus-Tumor Effect in Patients with Hematologic Malignancies Undergoing Allogeneic T Cell-Replete Haploidentical Transplantation

Followed by Post-Transplant Cyclophosphamide. *Biol Blood Marrow Transplant* (2018) 24:549–554. doi:10.1016/j.bbmt.2017.11.042

6. Shimoni A, Labopin M, Lorentino F, Van Lint MT, Koc Y, Gülbas Z, Tischer J, Bruno B, Blaise D, Pioltelli P, et al. Killer cell immunoglobulin-like receptor ligand mismatching and outcome after haploidentical transplantation with post-transplant cyclophosphamide. *Leukemia* (2019) 33:230–239. doi:10.1038/s41375-018-0170-5

7. Hsu KC, Keever-Taylor CA, Wilton A, Pinto C, Heller G, Arkun K, O'Reilly RJ, Horowitz MM, Dupont B. Improved outcome in HLA-identical sibling hematopoietic stem-cell transplantation for acute myelogenous leukemia predicted by KIR and HLA genotypes. *Blood* (2005) 105:4878–4884. doi:10.1182/blood-2004-12-4825

8. Nowak J, Kościńska K, Mika-Witkowska R, Rogatko-Koroś M, Mizia S, Jaskuła E, Polak M, Mordak-Domagała M, Lange J, Gronkowska A, et al. Donor NK cell licensing in control of malignancy in hematopoietic stem cell transplant recipients. *Am J Hematol* (2014) 89:E176-183. doi:10.1002/ajh.23802

9. Cooley S, Weisdorf DJ, Guethlein LA, Klein JP, Wang T, Le CT, Marsh SGE, Geraghty D, Spellman S, Haagenson MD, et al. Donor selection for natural killer cell receptor genes leads to superior survival after unrelated transplantation for acute myelogenous leukemia. *Blood* (2010) 116:2411–2419. doi:10.1182/blood-2010-05-283051

10. Symons HJ, Leffell MS, Rossiter ND, Zahurak M, Jones RJ, Fuchs EJ. Improved survival with inhibitory Killer Immunoglobulin Receptor (KIR) gene mismatches and KIR haplotype B donors after nonmyeloablative, HLA-haploidentical bone marrow transplantation. *Biol Blood Marrow Transplant* (2010) 16:533–542. doi:10.1016/j.bbmt.2009.11.022

11. Boudreau JE, Giglio F, Gooley TA, Stevenson PA, Le Luduec J-B, Shaffer BC, Rajalingam R, Hou L, Hurley CK, Noreen H, et al. KIR3DL1/ HL A-B Subtypes Govern Acute Myelogenous Leukemia Relapse After Hematopoietic Cell Transplantation. *J Clin Oncol* (2017) 35:2268–2278. doi:10.1200/JCO.2016.70.7059

12. Bari R, Rujkijyanont P, Sullivan E, Kang G, Turner V, Gan K, Leung W. Effect of Donor KIR2DL1 Allelic Polymorphism on the Outcome of Pediatric Allogeneic Hematopoietic Stem-Cell Transplantation. *J Clin Oncol* (2013) 31:3782–3790. doi:10.1200/JCO.2012.47.4007

13. Cook MA, Milligan DW, Fegan CD, Darbyshire PJ, Mahendra P, Craddock CF, Moss PAH, Briggs DC. The impact of donor KIR and patient HLA-C genotypes on outcome following HLA-identical sibling hematopoietic stem cell transplantation for myeloid leukemia. *Blood* (2004) 103:1521–1526. doi:10.1182/blood-2003-02-0438

14. Verheyden S, Schots R, Duquet W, Demanet C. A defined donor activating natural killer cell receptor genotype protects against leukemic relapse after related HLA-identical hematopoietic stem cell transplantation. *Leukemia* (2005) 19:1446–1451. doi:10.1038/sj.leu.2403839

15. Leung W, Iyengar R, Turner V, Lang P, Bader P, Conn P, Niethammer D, Handgretinger R. Determinants of antileukemia effects of allogeneic NK cells. *J Immunol* (2004) 172:644–650. doi:10.4049/jimmunol.172.1.644

16. Michaelis SU, Mezger M, Bornhäuser M, Trenschel R, Stuhler G, Federmann B, Oevermann L, Kanz L, Handgretinger R, Bethge WA. KIR haplotype B donors but not KIR-ligand mismatch result in a reduced incidence of relapse after haploidentical transplantation using reduced intensity conditioning and CD3/CD19-depleted grafts. *Ann Hematol* (2014) 93:1579–1586. doi:10.1007/s00277-014-2084-2

17. Hosokai R, Masuko M, Shibasaki Y, Saitoh A, Furukawa T, Imai C. Donor Killer Immunoglobulin-Like Receptor Haplotype B/x Induces Severe Acute Graft-versus-Host Disease in the Presence of Human Leukocyte Antigen Mismatch in T Cell-Replete Hematopoietic Cell Transplantation. *Biol Blood Marrow Transplant* (2017) 23:606–611. doi:10.1016/j.bbmt.2016.12.638

18. Solomon SR, Aubrey MT, Zhang X, Piluso A, Freed BM, Brown S, Jackson KC, Morris LE, Holland HK, Solh MM, et al. Selecting the Best Donor for Haploidentical Transplant: Impact of HLA, Killer Cell Immunoglobulin-Like Receptor Genotyping, and Other Clinical Variables. *Biol Blood Marrow Transplant* (2018) 24:789–798. doi:10.1016/j.bbmt.2018.01.013
